# Loss of Anti-Tumor Efficacy by Polyamine Blocking Therapy in GCN2 Null Mice

**DOI:** 10.3390/biomedicines11102703

**Published:** 2023-10-05

**Authors:** Eric T. Alexander, Erin Fahey, Otto Phanstiel, Susan K. Gilmour

**Affiliations:** 1 Lankenau Institute for Medical Research, 100 Lancaster Avenue, Wynnewood, PA 19096, USA; alexandere@mlhs.org (E.T.A.);; 2 Department of Medical Education, College of Medicine, University of Central Florida, Biomolecular Research Annex, 12722 Research Parkway, Orlando, FL 32826, USA; otto.phanstiel@ucf.edu

**Keywords:** polyamine blocking therapy, GCN2, α-difluoromethylornithine, transport inhibitor, macrophages, myeloid derived suppressor cells

## Abstract

GCN2 is one of the main sensors of amino acid starvation stress, and its activation in the stressful tumor microenvironment plays a crucial role in tumor survival and progression. We hypothesized that elevated polyamine biosynthesis and subsequent depletion of precursor arginine activates GCN2, thus rewiring metabolism to support tumor cell survival and drive myeloid immunosuppressive function. We sought to determine if the anti-tumor efficacy of a polyamine blocking therapy (PBT) may be mediated by its effect on GCN2. Unlike wild-type mice, PBT treatment in GCN2 knockout mice bearing syngeneic B16.F10 or EG7 tumors resulted in no tumor growth inhibition and no changes in the profile of infiltrating tumor immune cells. Studies with murine bone marrow cell cultures showed that increased polyamine metabolism and subsequent arginine depletion and GCN2 activation played an essential role in the generation and cytoprotective autophagy of myeloid derived suppressor cells (MDSCs) as well as the M2 polarization and survival of macrophages, all of which were inhibited by PBT. In all, our data suggest that polyamine-dependent GCN2 signaling in stromal cells promotes tumor growth and the development of the immunosuppressive tumor microenvironment, and that the PBT anti-tumor effect is mediated, at least in part, by targeting GCN2.

## 1. Introduction

Tumor development requires increased protein synthesis which can result in metabolite-related stress and a persistent state of nutrient starvation. A wide range of stressors in the tumor microenvironment can trigger the integrated stress response (ISR) that is mediated by four sensors: general control nonderepressible (GCN2) that detects amino acid depletion, protein kinase R (PKR) that recognizes viral double-stranded RNA, heme-regulated inhibitor (HRI) that is induced by heme deficiency, and PKR-like endoplasmic reticulum kinase (PERK) that detects ER stress. GCN2 is a Ser/Thr kinase that is activated in multiple solid tumors in response to metabolic endoplasmic reticulum (ER) stress such as amino acid deprivation [1,2,3]. Activation of GCN2 signaling promotes tumor cell survival via phosphorylation of eIF2α which leads to global reduction in cap-dependent translation and activation of ATF4 (activating transcription factor 4) [4,5,6,7]. In addition to ensuring metabolites to stressed cells by rewiring their metabolism, the GCN2-ATF4 pathway regulates oxidative stress by increasing glutathione levels and halts apoptosis [8,9,10]. Moreover, activation of GCN2 also increases the generation of myeloid derived suppressor cells (MDSC) and immune suppression in the tumor microenvironment [11,12]. In short, GCN2 is a major sensor that modulates the immune system and facilitates tumor survival under stress conditions.

In response to oncogene-driven anabolic metabolism in tumors, polyamine levels are dramatically elevated in tumors compared to normal tissue [13,14,15,16]. Polyamines (putrescine, spermidine, and spermine) are arginine-derived polycations that have been implicated in a wide array of biological processes, and they are essential for cellular proliferation, differentiation, and cell survival [14,17,18]. Intracellular polyamine levels are maintained via tightly regulated biosynthetic, catabolic, and uptake and export pathways [17,18]. Levels of the rate-limiting polyamine biosynthetic enzyme, ornithine decarboxylase (ODC), are elevated in many cancers [19], largely driven by oncogenes such as c-Myc [20,21]. In addition to driving polyamine biosynthesis, MYC also upregulates the cellular uptake of polyamines by inducing polyamine transport system (PTS) activity [22,23]. We hypothesized that elevated polyamine biosynthesis and subsequent depletion of the amino acid precursor arginine in the tumor microenvironment triggers the activation of GCN2, thus rewiring metabolism to support tumor cell survival and drive myeloid immunosuppressive function.

Previous clinical trials have tested the anti-tumor efficacy of α-difluoromethylornithine (DFMO, Eflornithine), an irreversible ODC inhibitor and FDA-approved drug for the treatment of *Trypanosomiasis* [24]. Although treatment with DFMO has had only moderate success in treating cancer patients [14,18,25], DFMO has gained new relevance as an adjunct therapy in children with high-risk neuroblastoma [26,27]. Subsequent studies discovered that DFMO-inhibition of ODC leads to upregulation of the polyamine transport system (PTS) with resulting increased uptake of polyamines from the diet and gut flora into the tumor cells [13]. To polyamine-starve a tumor (and limit its progression), we have developed a polyamine blocking therapy (PBT) that includes a combination of DFMO and a novel polyamine transport inhibitor (PTI), i.e., a three-armed-polyamine compound, Trimer44NMe PTI [28]. By blocking both polyamine biosynthesis and polyamine import, PBT reduces polyamines in the tumor microenvironment and suppresses tumor growth in multiple animal tumor models [16,29]. In this report, we use two murine tumor models in which GCN2 is knocked out to investigate if the anti-tumor effect of PBT is mediated by its effect on GCN2 signaling in stromal cells in the tumor microenvironment. 

## 2. Methods

### 2.1. Cell Line Culture

B16F10-sTAC melanoma cells, which are B16F10 cells engineered to express the SIINFEKL peptide [16], were cultured in DMEM supplemented with 10% fetal bovine serum and 1× Penicillin/Streptomycin. EG7 lymphoma cells were cultured in RPMI supplemented with 10% heat inactivated fetal bovine serum, 1× Penicillin/Streptomycin, 50 µM β-mercaptoethanol, and 0.4 mg/mL G418. Cell line authentication and IMPACT tests were performed on these cells before use in animal experiments. The cells were freshly thawed from early passage cells, cultured for no more than 2 months, and regularly checked by virtue of their morphologic features to avoid cross-contamination or misuse.

### 2.2. Animals 

Female C57Bl/6 mice were obtained from Charles Rivers/NCI, and GCN2 null mice on a C57Bl/6 background (Strain #:008240) were obtained from Jackson Laboratory. Protocols for the use of animals in these studies were reviewed and approved by the Institutional Animal Care and Use Committee of the Lankenau Institute for Medical Research in accordance with the current US Department of Agriculture, Department of Health, and Human Service regulations and standards.

### 2.3. Drugs

Working solutions of the ODC inhibitor DFMO (kindly donated by Dr. Patrick Woster at the University of South Carolina) were prepared in sterile water. The Trimer PTI (N1,N1′,N″-(benzene-1,3,5-triyltris(methylene))tris(N4-(4-(methylamino)butyl)butane-1,4-diamine) was synthesized as previously described [28], and 1 mM stock solutions were prepared in sterile PBS and stored at −20 °C. Chemical authentication of the Trimer PTI included ^1^H NMR, ^13^C NMR, mass spectrometry, and elemental analysis of the final compound used in this study. This information matched the literature data for Trimer PTI.

### 2.4. Bone Marrow-Derived Macrophages

For macrophage cultures, bone marrow was flushed from the femurs of C57BL/6 mice with RPMI under sterile conditions, red blood cells lysed in lysis buffer (0.17 M Tris-HCL, 0.16 M NH_4_Cl) for 3 min, and then resuspended in bone marrow media (RPMI, 10% FBS and 1% penicillin/streptomycin). Cells were plated in 6 well dishes with 2 mL/well bone marrow media containing 20 ng/mL macrophage colony stimulating factor (M-CSF) (R&D Systems, Minneapolis, MN, USA, 216MC025). On day two, an additional 2 mL of bone marrow media containing 20 ng/mL M-CSF was added. On day five, 2 mL of media was gently removed and 2 mL of bone marrow media containing 20 ng/mL M-CSF was added. By day 7, adherent cells had differentiated into macrophages. Macrophages were polarized to M1 or M2 with 100 ng/mL LPS (Sigma, Saint Louis, MO, USA, L2630) or 10 ng/mL IL-4 (R&D Systems, 404-ML-010), respectively [30]. For experiments with arginine-deficient media, the base RPMI was replaced with arginine-deficient RPMI (Thermo Scientific 88365, Waltham, MA, USA).

### 2.5. Bone Marrow-Derived MDSCs

For MDSC cultures, bone marrow was flushed from the femurs of C57BL/6 mice with RPMI under sterile conditions, red blood cells lysed in lysis buffer (0.17 M Tris-HCL, 0.16 M NH_4_Cl) for 3 min, and then resuspended in bone marrow media (RPMI, 10% FBS and 1% penicillin/streptomycin). Bone marrow cells were then plated in 6-well plates and cultured in bone marrow media + 60 ng/mL IL-6 and 60 ng/mL GM-CSF for up to 72 h [31]. For experiments with arginine deficient media, the base RPMI was replaced with arginine deficient RPMI (Thermo Scientific 88365).

### 2.6. Flow Cytometry Analysis of Immune Cells

Tumor tissue was digested in a 0.3% collagenase/0.1% hyaluronidase solution, pressed through a nylon mesh filter to obtain a single cell suspension and incubated in red cell lysis buffer (0.17 M Tris-HCl, 0.16 M NH_4_Cl) for 3 min, spun down, and resuspended in FACS buffer (PBS + 1.5% FBS). Equal numbers of cells were stained with a viability dye and combinations of the following antibodies: CD8a-PECy7 (eBioscience, San Diego, CA, USA, 25-0081-82), F4/80-PECy7(eBioscience, 25-4801-82), CD206-FITC (Biolegend, 141704), Ly6G-APC (eBioscience, 17-5931-81), CD11b-PE (eBioscience, 12-0112-82), CD45-PE-Cy5 (eBioscience, 15-0451-83), IFN-γ-APC (eBioscience, 17-7311-82), and SIINFEKL pentamer-PE (ProImmune, Oxford, UK). For autophagy analysis using Cyto-ID staining of bone marrow derived macrophages, cells were first stained for surface markers (CD80-PE-Cy5 (eBioscience, 15-0801-81) and CD206-APC (eBioscience, 17-2061-82)) followed by staining with Cyto-ID for 30 min at 37 °C, per the manufacturer’s protocol (Enzo Life Sciences, Farmingdale, NY, USA, ENZ-51031). Flow cytometric data were acquired on a BD FACSCanto II cytometer and analyzed using FACSDiva software version 9.2 (BD Biosciences, San Jose, CA, USA). 

### 2.7. In Vivo Tumor Models

Tumor models were established by subcutaneous injections of 5 × 10^5^ B16F10-sTAC melanoma cells or 1 × 10^6^ EG7 lymphoma cells in C57Bl/6 mice. Treatment with 0.5% (*w*/*v*) DFMO in the drinking water and Trimer PTI (3 mg/kg daily by intraperitoneal [i.p.] injection) was initiated when tumors were palpable (50–100 mm^3^). Tumor growth was assessed morphometrically using calipers three times a week, and tumor volumes were calculated using the formula V (mm^3^) = π/6 × A × B^2^ (A is the larger diameter and B is the smaller diameter) [32]. 

### 2.8. Antigen-Specific T-Cell Response Detection by IFN-γ ELISpot

Upon sacrifice, splenocytes from B16F10-sTAC tumor-bearing mice were analyzed for IFN-γ producing cells by enzyme-linked immunosorbent spot (ELISpot) assay. Multiscreen filtration plates (Millipore, Burlington, MA, USA, MSHAN4550) were coated with 0.5 µg/mL of purified anti-mouse IFN-γ capture antibody (Biolegend, San Diego, CA, USA, 505702) overnight at 4 °C. Single-cell suspensions of splenocytes or tumors were plated at 1 × 10^6^ per well. Splenocytes were stimulated with the SIINFEKL peptide (Anaspec, Fremont, CA, USA, AS-60193-1) at 20 µg/mL. After 16 h of stimulation at 37 °C, the cells were removed by washing and spots were developed with a biotinylated anti-IFN-γ detection antibody (Biolegend, 505804) and streptavidin-horseradish peroxidase conjugate followed by NITRO-blue tetrazolium chloride and 5-bromo-4-chloro-3′-indoylphosphate p-toluidine salt substrate (Sigma, B5655-25TAB). Spot numbers were counted, and data were reported as IFN-γ-spot forming cells per 10^6^ cells.

### 2.9. Quantitative PCR

RNA was isolated from MDSCs using Trizol (Life Technologies, Carlsbad, CA, USA, 15596026) and converted to cDNA using the GoScript Reverse Transcription System (Promega, Madison, WI, USA, A5000). Primers for IL-6 (AGCCAGAGTCCTTCAGAGA and TCCTTAGCCACTCCTTCTGT), ARG1 (GAATGGAAGAGTCAGTGTGGT and AGTGTTGATGTCAGTGTGAGC), and the housekeeping gene B2M (GGACTGGTCTTTCTATCTCTTGT and ACCTCCATGATGCTGCTTAC) were purchased from IDT and used in conjunction with SYBR Green JumpStart Taq ReadyMix (Sigma-Aldrich, S44388-500RXN) on a QuantStudio 3 (Applied Biosystems, Waltham, MA, USA).

### 2.10. Statistical Analysis

All in vitro experiments were performed at least in triplicate, and data were compiled from two to three separate experiments. Analyses were performed using a 1-way ANOVA with a Tukey test for statistical significance or a Student’s t-test test to assess the statistical significance among groups using GraphPad Prism software (v8; GraphPad Software, Inc., La Jolla, CA, USA. In vivo studies were carried out using multiple animals (*n* = 5–10 per treatment group based on previous repeated murine tumor experiments). The mean profile plots were created in Stata/MP 15.1 (StataCorp LP., College Station, TX, USA) and all other analyses were performed in SAS 9.4 SAS/STAT Version 14.1 for Windows (SAS Institute Inc., Cary, NC, USA). All tests were two-sided, and the statistical significance level was set to 0.05. Tumor growth curves were analyzed with a generalized linear model with fixed effects of treatment and time. Data were examined for the interaction between treatment groups and day of observations, testing whether the slopes of the growth curves (tumor volume vs. day of observation) were significantly different for the control and treatment groups. In all cases, values of *p* < 0.05 were regarded as being statistically significant.

## 3. Results

**Figure 1:**
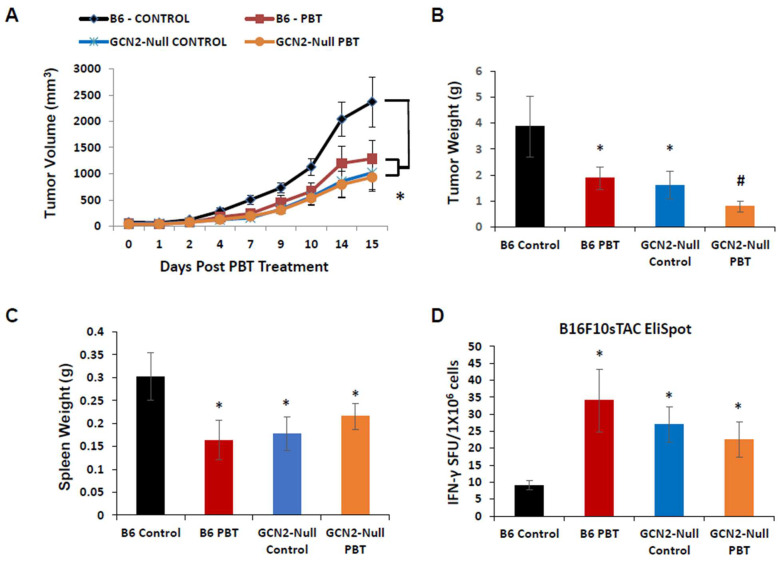
Requirement of GCN2 in the tumor microenvironment for PBT inhibition of B16F10-sTAC tumor growth. (**A**) C57Bl/6 and GCN2-null mice were subcutaneously injected with 5 × 10^5^ B16F10-sTAC melanoma cells (GCN2 WT). When tumors were 50–100 mm^3^ in size, treatment was initiated with 0.5% DFMO (*w*/*v*) in the drinking water and Trimer PTI (3 mg/kg, i.p., daily). Graph shows B16F10-sTAC tumor growth for mice in different treatment groups (mean tumor volume ± SEM). (**B**) Upon sacrifice, tumors were excised and weighed (mean ± SEM). (**C**) Upon sacrifice, spleens were excised and weighed (mean ± SEM). (**D**) The frequency of IFN-γ producing T-cells was measured by the ELISpot assay as IFN-γ spot forming units (SFU) per million cells. n = 5–10 mice per group. * = *p* ≤ 0.05, # = *p* ≤ 0.01 compared to B6 controls.

Because polyamine blocking therapy (PBT) was shown to significantly inhibit tumor growth and reduce the immunosuppressive tumor microenvironment [16,29], we used GCN2^−/−^ mice to investigate if polyamine-dependent signaling through the GCN2 receptor via arginine depletion plays a role in the development of the immunosuppressive tumor microenvironment. To evaluate this, we used the B16F10-sTAC melanoma model which is GCN2^+/+^. Following subcutaneous injection of B16F10-sTAC cells, which are B16F10 cells that have been engineered to express the SIINFEKL peptide, in C57Bl/6 and GCN2^−/−^ mice, treatment was initiated when the resulting tumors were between 50 and 100 mm^3^ in size. Mice were administered Trimer PTI (i.p. injection, 3 mg/kg daily) with 0.5% DFMO (*w*/*v*) in the drinking water. As was previously shown [16], there was a significant inhibitory effect on tumor growth in C57Bl/6 mice treated with both Trimer PTI and DFMO (Figure 1A,B). Tumor growth in GCN2-null mice was significantly inhibited compared to that in C57Bl/6 mice demonstrating the role of GCN2 in the tumor microenvironment in tumor growth. Interestingly, this growth inhibition in GCN2 null mice was comparable to PBT-treated C57Bl/6 mice (Figure 1A,B). Importantly, treatment of GCN2-null mice with PBT resulted in no further inhibition of tumor growth suggesting that PBT may work via GCN2 signaling. We also noted that C57Bl/6 mice treated with PBT and GCN2-null mice had reduced spleen weights compared to control C57Bl/6 mice (Figure 1C). PBT treatment did not further reduce spleen weight in GCN2-null mice compared to the untreated control GCN2-null mice.

**Figure 2:**
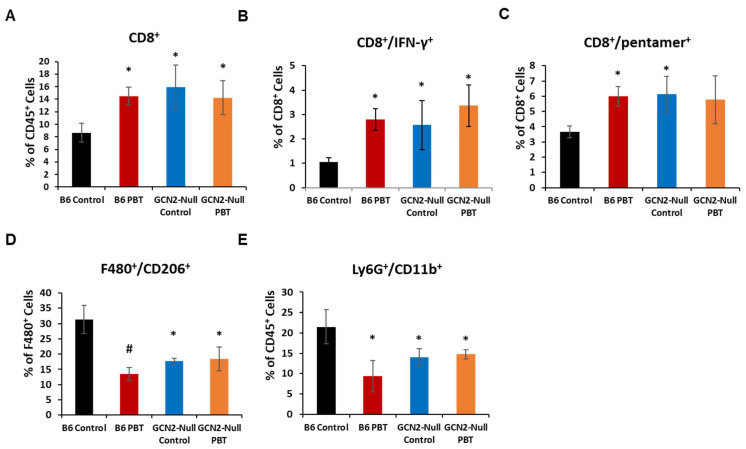
Inhibition of GCN2 signaling alters infiltrating lymphocyte cell populations. Upon sacrifice, tumors were excised from B16F10-sTAC-tumor bearing mice and processed for flow cytometry. Cells were analyzed for the percentage of the indicated cell subpopulations. n = 5–10 mice per group. * = *p* ≤ 0.05, # = *p* ≤ 0.01 compared to B6 controls.

The decreased tumor growth in C57Bl/6 mice treated with DFMO and Trimer PTI was associated with a significant increase in IFN-γ producing splenocytes as measured by the ELISpot assay following ex vivo stimulation with the SIINFEKL peptide (Figure 1D). GCN2-null mice also demonstrated a significant increase in IFN-γ producing splenocytes compared to C57Bl/6 control mice but the addition of PBT treatment in GCN2-null mice did not further increase the number of IFN-γ splenocytes. The increase in IFN-γ producing splenocytes in PBT-treated C57Bl/6 mice and control GCN2-null mice was mirrored in the tumor where total CD8^+^ T-cells and CD8^+^/IFN-γ^+^ T-cells were increased compared with control C57Bl/6 tumor-bearing mice (Figure 2A,B). Similarly, PBT treatment in GCN2-null mice did not further increase intratumoral CD8^+^ T-cells and CD8^+^/IFN-γ^+^ T-cells compared to control GCN2-null mice. Pentamer analysis of the SIINFEKL recognition site on CD8^+^ cytotoxic T-cells showed that C57Bl/6 mice treated with PBT and GCN2-null mice, with and without PBT, had significantly higher frequencies of SIINFEKL-specific CD8^+^ T-cells compared to control C57Bl/6 mice (Figure 2C). Compared with control C57Bl/6 mice, C57Bl/6 mice receiving PBT and GCN2-null mice, with and without PBT, also had significantly reduced levels of immunosuppressive F480^+^/CD206^+^ M2 macrophages and Ly6G^+^/CD11b^+^ MDSCs. These changes in immune cell frequency, increases in tumor specific cytotoxic T-cells and reductions in immunosuppressive macrophages and MDSCs, with PBT treatment in C57Bl/6 mice or in GCN2-knockouts suggested that polyamine-dependent signaling through GCN2 is playing a key role in regulating the immune response to the tumor.

**Figure 3:**
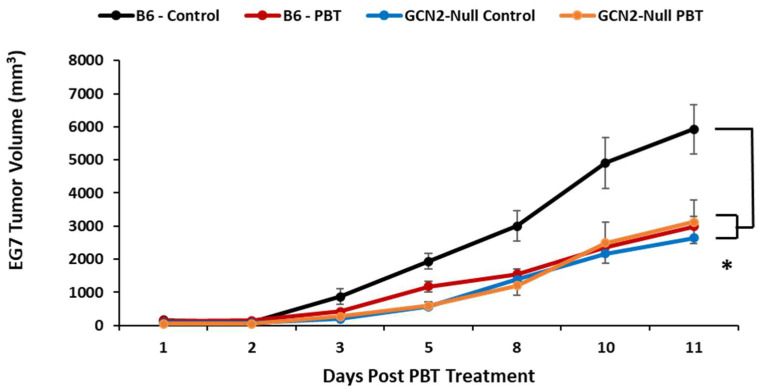
Requirement of GCN2 in the tumor microenvironment for PBT inhibition of EG7 tumor growth. C57Bl/6 and GCN2-null mice were subcutaneously injected with 1 × 10^6^ EG7 lymphoma cells (GCN2 WT). When tumors were 50–100 mm^3^ in size, treatment was initiated with 0.5% DFMO (*w*/*v*) in the drinking water and Trimer PTI (3 mg/kg, i.p., daily). Graph shows EG7 tumor growth for mice in different treatment groups (mean tumor volume ± SEM). * = *p* ≤ 0.05, compared to B6 controls.

The observations made in the B16F10sTAC tumor model system were confirmed with the EG7 lymphoma tumor model which is also GCN2^+/+^. Following subcutaneous injection of EG7 cells in C57Bl/6 and GCN2^−/−^ mice, PBT treatment was initiated when the resulting tumors were between 50 and 100 mm^3^ in size. Mice were administered Trimer PTI (i.p. injection, 3 mg/kg daily) with 0.5% DFMO (*w*/*v*) in the drinking water. There was a significant inhibitory effect on tumor growth in C57Bl/6 mice treated with PBT (Figure 3). In GCN2-null mice, tumor growth was significantly inhibited compared to C57Bl/6 mice and was comparable to C57Bl/6 mice which received PBT (Figure 3). Treatment of GCN2-null mice with PBT did not further inhibit tumor growth compared to vehicle-treated GCN2-null mice.

**Figure 4:**
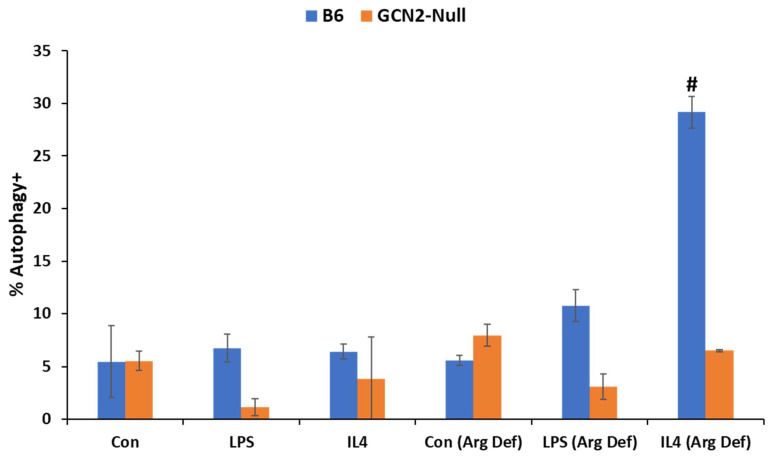
Requirement of GCN2-signaling to trigger cytoprotective autophagy in immunosuppressive M2 macrophages. Bone marrow-derived macrophages from C57Bl/6 or GCN2 null mice were polarized to M2-like macrophages with IL-4 treatment or to M1-like macrophages with LPS treatment in either complete or arginine-deficient (Arg Def) media for two days. Macrophages were analyzed by flow cytometry for the percentage of autophagy^+^ macrophages following staining with Cyto-ID. n = 3 per group ± SD. # = *p* ≤ 0.01 compared to respective controls incubated with complete media.

We have previously shown that under the harsh conditions of the tumor microenvironment, tumor-infiltrating immunosuppressive myeloid cell populations such as MDSCs and M2-macrophages undergo elevated levels of cytoprotective autophagy which facilitate their survival and pro-tumorigenic activities [29]. Polyamine metabolism plays a key role in this process with administration of PBT dramatically reducing levels of autophagy in tumor-infiltrating immunosuppressive myeloid cells compared to control cells [29]. We hypothesized that elevated polyamine biosynthesis and subsequent depletion of the polyamine precursor arginine activates GCN2, rewiring cell metabolism, and promotes cytoprotective autophagy. To investigate this, macrophages were differentiated in vitro from bone marrow cells from C57Bl/6 and GCN2-null mice using M-CSF. When the macrophages were treated with LPS or IL-4 to promote polarization to the CD80^+^ proinflammatory, antitumor (M1) or the CD206^+^ immunosuppressive, protumorigenic (M2) states, there was no difference in the degree of polarization between C57Bl/6 and GCN2-null macrophages (Supplemental Figure S1). Using a flow cytometry-based assay with the fluorescent dye Cyto-ID that colocalizes with LC3 on autophagosomes, we assessed the levels of autophagic flux in macrophages following polarization in complete or arginine-deficient media. Bone marrow-derived macrophages isolated from B6 mice and polarized to the M2 state with IL-4 showed increased autophagic flux when incubated in arginine-deficient media. However, bone marrow-derived macrophages from GCN2-null mice demonstrated no increase in autophagy when incubated with arginine-deficient media (Figure 4). Incubation in arginine-deficient media had no effect on macrophage polarization markers (CD80 and CD206) in cells treated with either LPS or IL-4 (Supplemental Figure S1).

**Figure 5:**
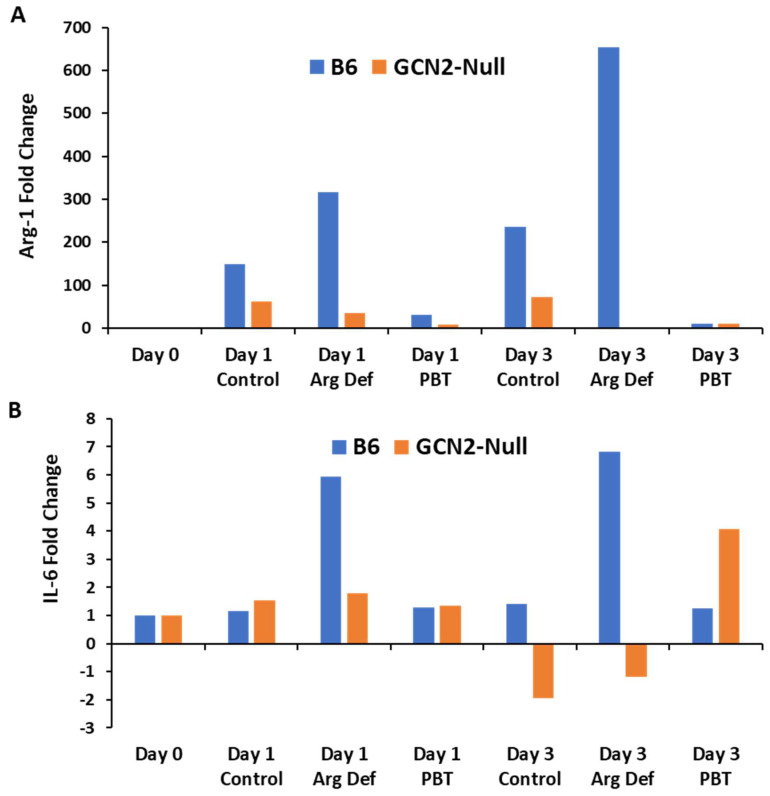
Promotion of an immunosuppressive phenotype in MDSCs by GCN2-signaling. Bone marrow-derived MDSCs from C57Bl/6 or GCN2-null mice were incubated in either complete or arginine-deficient (Arg Def) media for one or three days at which point RNA was isolated and used to perform qPCR to measure (**A**) Arg-1 and (**B**) IL-6 gene expression levels. Data expressed as fold change compared to Day 0 when the bone marrow cells were initially cultured. These data suggest that both GCN2 and PBT influence arginase-1 mRNA expression. Representative of three experiments with similar results.

Because arginine depletion led to a strong upregulation of cytoprotective autophagy in wild-type immunosuppressive M2 macrophages but not in proinflammatory M1 macrophages (Figure 4), we hypothesized that arginine depletion due to increased polyamine biosynthesis may have profound effects on other immunosuppressive cell types in the tumor microenvironment. To investigate this hypothesis, we differentiated MDSCs using bone marrow cells from C57/Bl/6 and GCN2-null mice using complete or arginine-deficient medias containing GM-CSF and IL-6. After one or three days of differentiation, RNA was isolated from MDSCs and used for quantitative PCR. Arginase 1 gene expression levels dramatically increased over time in C57Bl/6 derived MDSCs especially after culture in arginine-deficient media, but not in GCN2-null MDSCs (Figure 5A). Arginase 1 activity is associated with the ability of MDSCs to inhibit cytotoxic T-cell activity due to the depletion of arginine, thus facilitating tumor immune escape [33]. Incubation with PBT completely blocked the upregulation of arginase 1 expression in both B6 and GCN2-null macrophages. The dramatic upregulation of arginase 1 in C57Bl/6-derived MDSCs incubated in arginine-deficient media may be due, in part, to increased IL-6 expression, which has been shown to upregulate arginase 1 in MDSCs [34] but not in GCN2-null derived MDSCs (Figure 5B). We note that GCN2 was previously shown to be required for IL-6 upregulation in myeloid cells in response to amino acid deprivation via a CHOP-dependent signaling mechanism [35]. Overall, these data suggest that polyamine-dependent GCN2 signaling in stromal cells promotes tumor growth and the development of an immune-suppressive tumor microenvironment, and that PBT anti-tumor efficacy is mediated, at least in part, by its effect on GCN2 signaling in the tumor microenvironment. 

## 4. Discussion

Previous studies have demonstrated the dependency on T-cells for the anti-tumor effect of PBT [16,36]. Here, we demonstrate that the anti-tumor efficacy of PBT also depends upon its inhibition of GCN2 activation in stromal cells. Multiple studies have demonstrated a key role of the ISR, including GCN2 activation, in response to stressful environmental changes such as amino acid depletion and restoring cellular homeostasis. The tumor microenvironment is usually nutrient poor due to insufficient tumor blood perfusion and increased metabolic demand in the tumor. GCN2 is activated in multiple tumor types in response to amino acid starvation in order to support tumor cell survival [4]. Activation of the GCN2-eIF2α-ATF4 pathway regulates a number of genes involved in amino acid import and synthesis as well as cytoprotective autophagy. Similar to previous reports [11], we found that tumor growth was reduced in GCN2 null mice compared to that in wild-type mice. Moreover, the anti-tumor efficacy of PBT was lost in GCN2 null mice, suggesting that blockade of polyamines inhibits GCN2 activation that is necessary for tumor survival. 

Since GCN2 was only knocked out in stromal cells (including immune cells, fibroblasts, and endothelial cells) but not in the tumor cells, our attention turned to the effect of GCN2 on infiltrating immune cells. Perturbations of polyamine metabolism can impact arginine metabolism within the tumor microenvironment particularly because polyamine biosynthesis depends on arginase that supplies ornithine to ODC. An important determinant of the inflammatory response of macrophages and dendritic cells is the balance of arginine metabolism via nitric oxide synthase (NOS) or arginase. Immunosuppressive tumor-infiltrating immune cells, including MDSCs, M2-like macrophages, immature dendritic cells, and Tregs, are all characterized by elevated levels of arginase and the ability to profoundly suppress T-cell functions via arginase-mediated depletion of arginine [37,38]. Increased L-arginine catabolism leads to GCN2 activation [11,39]. As part of this stress response, GCN2-driven autophagy is an important negative regulator of inflammatory stress and has been shown to promote the survival of immunosuppressive cells such as MDSCs [40,41,42]. In addition, autophagy has been shown to regulate the differentiation of macrophages into pro-tumorigenic TAMs [43,44], and GCN2 activation is required for MDSC generation and survival leading to increased tumor growth and progression [11]. 

The high demand in tumors for amino acids and polyamines to sustain their increased metabolism, protein synthesis, and proliferation results in nutrient limitations that activate GCN2. Induction of GCN2 signaling pathways promotes and sustains the generation of immunosuppressive myeloid cells that are critical for tumor growth and development. Thus, GCN2 activation induces a set of genes responsible for maintaining amino acid homeostasis that are essential for tumor progression. Conversely, loss or inhibition of GCN2 function slows tumor growth and presents a vulnerability for many tumors that can be targeted therapeutically. By using GCN2 null mice in which GCN2 is knocked out only in stromal cells but not in the tumor cells, we were able to demonstrate the importance of the presence of this stromal sensor to respond to changes in the tumor microenvironment that support tumor growth. We observed that under arginine-deficient conditions, GCN2 activation is essential for the generation of MDSCs and the survival of M2-like macrophages via cytoprotective autophagy, all of which helps the tumor survive. Our data suggest that polyamine deficient conditions induced by PBT treatment mitigates activation of GCN2 signaling pathways that enable tumors to adapt to microenvironmental stress inherent in all tumors and to escape immune surveillance, thus leading to decreased tumor growth and decreased tumor survival. Importantly, PBT inhibition of GCN2-generation and support of immunosuppressive macrophages and MDSCs supports its potential to be used to improve sensitivity to immunotherapy and standard chemotherapy in cancer patients.

## 5. Conclusion

Our data suggest that polyamine-dependent GCN2 signaling in stromal cells promotes tumor growth and the development of the immunosuppressive tumor microenvironment, and that the PBT anti-tumor effect is mediated, at least in part, by targeting GCN2.

## Data Availability

Data available upon request to S.K.G.

## Supplementary Materials

The following supporting information can be downloaded at: https://www.mdpi.com/article/10.3390/biomedicines11102703/s1, Figure S1. Arginine deficiency has no effect on macrophage polarization Bone.
