# Supplementary material for: Loss of Anti-Tumor Efficacy by Polyamine Blocking Therapy in GCN2 Null Mice
Source: Biomedicines. 2023 Oct 5;11(10):2703. doi: 10.3390/biomedicines11102703 (PMC10604246; doi:10.3390/biomedicines11102703)
Supplement: Supplementary file 1 [file biomedicines-11-02703-s001.zip › biomedicines-2545180-supplementary.pdf]

Sup. Fig. S1

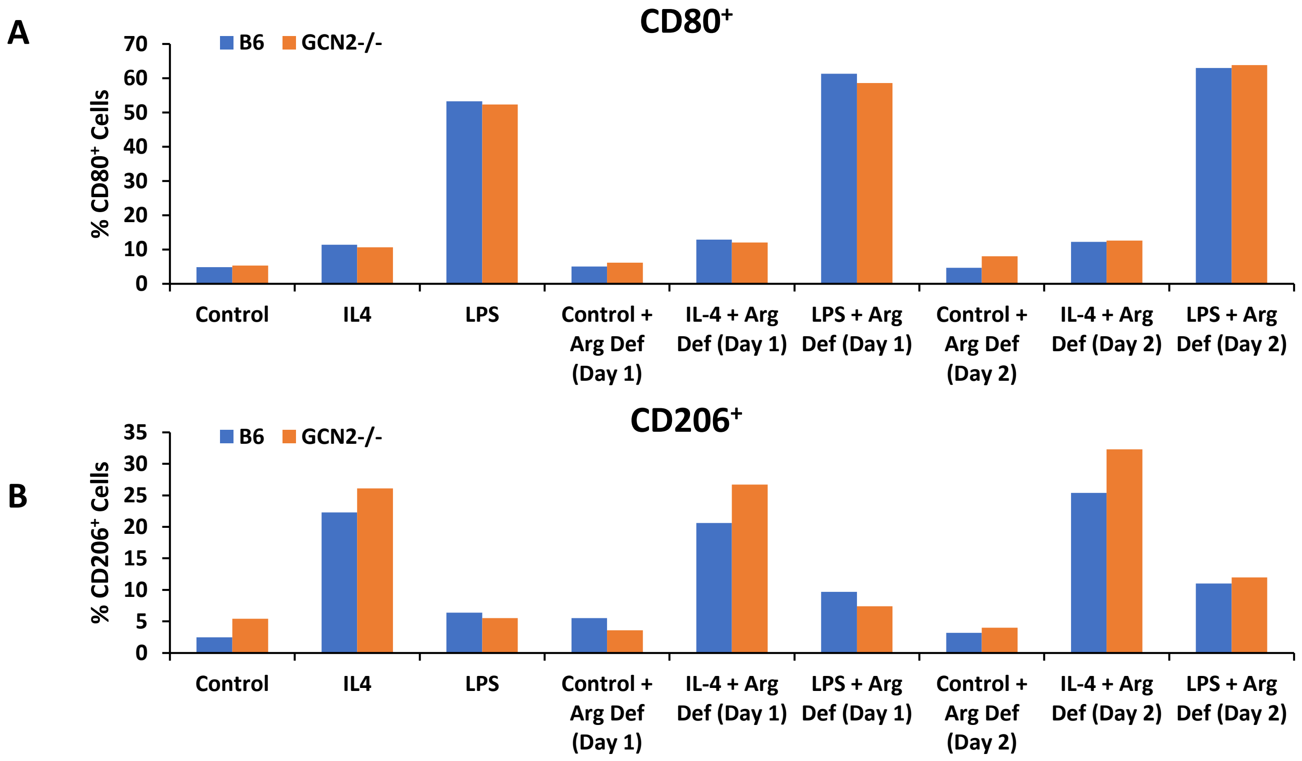

Supplementary Figure S1: Arginine deficiency has no effect on macrophage polarization. Bone marrow-derived macrophages from C57Bl/6 or GCN2 null mice were polarized to M2-like macrophages with IL-4 treatment or to M1-like macrophages with LPS treatment in either complete or arginine-deficient (Arg Def) media for one or two days. Macrophages were analyzed by flow cytometry for (A) $CD80^{+}$ M1 polarized macrophages, (B) $CD206^{+}$ M2 polarized macrophages
